# The stress of studying in China: primary and secondary coping interaction effects

**DOI:** 10.1186/s40064-015-1540-3

**Published:** 2015-12-02

**Authors:** Alexander S. English, Zhi Jia Zeng, Jian Hong Ma

**Affiliations:** Department of Psychology and Behavioral Sciences, Zhejiang University, Xixi Campus, Hangzhou, 310011 China

**Keywords:** Acculturative stress, Perceived cultural distance, Primary and secondary coping, Socio-cultural adaptation, Educational sojourners in China

## Abstract

Answering the call for research on coping outside of the Western world, the present study confirms previous research that indicated Asians cope with stress differently from other ethnic groups. In the present study, we explore the stress-coping-adjustment model and its role in acculturation for educational sojourners in the People’s Republic of China. Using a sample of 121 recent exchange students (Asian, *n* = 52; non-Asian, *n* = 69), we administered surveys in the Fall of 2013 and 90 days later to measure students’ socio-cultural adaptation. Results indicate several significant findings. Acculturative stress and perceived cultural distance had no role in predicting adaptation. Non-Asians reported greater adjustment even though cultural distance was greater. As hypothesized, non-Asians used more primary and secondary coping compared to Asians. Moderation analyses indicate three-way interactions among stress × coping × group, showing that non-Asians benefit from high usage primary coping, while primary coping exacerbates the negative effects of stress on adjustment for Asians. Secondary coping proves to be beneficial for both groups and improves adjustment across low and moderate stress levels. Results support recent developments in collective coping, suggesting that primary and secondary coping may not be beneficial for all ethnic groups in all circumstances. Research implications and practical contributions are discussed.

## Background

Chinese emigrants have a long history of establishing so-called “Chinatowns” around the world, but since the “Rise of China” epitomized by the 2008 Summer Olympics in Beijing, the influx of expats casts new light on a country that was closed off to the world for much of the 20th Century. Yearly, over 300,000 foreign students make their way to Mainland China to attend higher education institutes and by the year 2020, China will attract over half-a-million international students annually (Hennock, 2012). Students from the United States (27,000), Europe (35,000), South Korea (65,000), and Africa (21,000) study Mandarin Chinese or pursue degrees in China (Bodomo 2014; Hashim and Zhiliang 2003; Hui 2014; Hu 2012; Lin 2009). With the population of foreign students growing in sync with China’s booming economy, there should be no doubt for the need for more empirical cross-cultural studies on this population. Therefore in the present research, we hope to answer the question: how are foreign students in China adapting socially and culturally? How are they adjusting with the stress of learning Mandarin Chinese, being away home, learning a new culture, and living in an environment that is different from what they may be accustomed to.

Globalization and cross-cultural interactions are an integral part of a world where one out of every 33 people on earth is a migrant (International Organization for Migrants 2013). *Acculturation*, or “those phenomena which result when groups of individuals having different cultures come into continuous first-hand contact, with subsequent changes in the original culture patterns of either or both groups” (Redfield et al. 1936, p. 149), has become a key research concept for the past half century (Demes and Geeraert 2013). The individuals who move to a new culture, *sojourners*, do not always experience smooth and successful acculturation. In fact, acculturative stress has been long documented as an important factor affecting intercultural transitions (Ward and Rana-Deuba 1999).

## Acculturative Stress

*Acculturative stress*, which is rooted in the acculturation process according to Berry (1997, 2005, 2006), is the difficulty one faces while transitioning into a new culture (Smart and Smart 1995). Particular examples include not being able to communicate with locals, being uprooted from home, feelings of isolation, being powerless, and depression (Szabo et al. in press). Acculturative stress can depend on the type of migration (e.g. educational sojourner vs. economic migrant; see Boski, 2013) and be classified as intercultural, interpersonal, intrapersonal, academic, and environmental (Hashim and Zhiliang 2003). Acculturative stress has been linked to outcomes in acculturation such as one’s emotional well-being (*psychological adaptation*), health conditions (*health*-*related adaptation*), or behavioral competence in the new culture (*social*-*cultural adaptation*) (Nguyen and Benet-Martínez 2012).

## Coping and adaptation

*Coping* acts as a mechanism for individuals to restore a sense of control in their environment in response to a stressful experience. While coping models have been described by problem-focused vs. emotion-focused or engagement vs. disengagement, coping strategies have also been explained with the terms primary vs. secondary (Skinner et al. 2003). When a stress-provoking situation is encountered, individuals may exert a *primary coping* strategy aimed at taking direct action or behavioral modification to alter stress; or use a *secondary coping* strategy where one cognitively changes perceptions or reappraises the meaning of stressful events to recover from a stress-inducing event (see Rothbaum et al. 1982). In other words, primary coping aims to actively control stress by initiating a change in the ‘person-to-environment’ relationship (Folkman et al. 1986), while secondary coping aims to eliminate stress through an inward-directed process such as positive reinterpretation or acceptance (as explained in Szabo et al. in press). Although coping is not limited to acculturative stress, it is considered the key element in managing stress since its theoretical inception in research by Lazarus and Folkman (1984) and later supported by Carver et al. (1989).

## Cultural differences in coping

Researchers argue that a “one-size fits all” approach to evaluating coping should be avoided because western coping strategies are not applicable to the various ethnic groups across all regions of the world (Heppner 2008). To clarify, primary and secondary coping strategies have been shown to differ by cultural context and by the effectiveness across certain ethnic groups. Cross-cultural studies have investigated primary and secondary coping across different cultural backgrounds as in: Sinha and Watson (2007), comparing Canadians to Indians; Jose et al. (1994), comparing American and Russian early adolescents; McCarty et al. (1999), comparing Thai and American elementary school children and Tweed et al. (2004a, b), comparing East Asians to Canadians. The literature consistently supports the notion that various ethnic groups manage stress using different coping strategies. Among American college students in California, Lam and Zane (2004) found that Asian Americans used secondary coping more often than European Americans, while Euro-Americans used more primary coping. Secondary coping seems to be more strongly associated with Asian ethnic groups as opposed to Western (or non-Asian) groups, however one recent study found that Asians (Japanese students) may in fact prefer primary controls (see recent study by Sawaumi et al. 2015).

## Coping and the host environment

Despite the possible need to immerse oneself in a new culture, during acculturation, individuals may continue to employ coping strategies similar to how they would in their culture of origin. Selmer (2002) found that western expats in China use more problem-focused (primary coping) compared to Asian expats, while Asians used more secondary coping. However, it is still uncertain exactly which coping strategies benefit which ethnic groups in which contexts (see the review by Kuo 2014). In certain environments and situations, primary and secondary coping have been found to be beneficial and effective for alleviating psychological distress (see Chataway and Berry 1989; Kuo and Roysicar 2006; Noh and Kaspar 2004; Yoshihama 2002; Zheng and Berry 1991). Ward and Kennedy (2001) found that primary coping (active and planning strategies) predicted lower levels of depression in British expats in Singapore. These lines of study support the notion that individualistic societies^1^ use more primary coping even when acculturating to Eastern culture contexts. Selmer (2002) also suggested that collectivist societies use more emotion-focused (secondary) coping. However, Selmer (2002), Ward et al. (2001) and many other cross-sectional studies lack a connection to adjustment. To be more precise, no cross-cultural longitudinal study has explored stress-coping-adjustment relationship in Asia. Researchers continue to express the need for these studies to be conducted in under researched parts of the globe, but few have surfaced (Heppner 2008; Aldwin 2007; Chun et al. 2006; Wong and Wong 2006). The present study is first to be conducted in such a setting.

## Comparing two cultures

Cultures can often be compared by artifacts, cultural values and other aspects that are visible during cross-cultural interactions. In line with this notion is *cultural distance*, which is defined as distance between two cultures and is viewed as an important aspect in acculturation research (see Babiker et al. 1980). Various researchers have found evidence to support that greater cultural distance leads to poorer socio-cultural adaptation and/or more psychological maladjustment (see Searle and Ward 1990; Ward et al. 2001; Ward and Kennedy 1999; Galchenko and van de Vijver 2007). In parallel with objective measures of cultural distance (e.g. GDP, GINI, Hofstede’s and Schwartz’ cultural dimensions), is the subjective measure of *perceived cultural distance* (PCD) measured by one’s actual observed measure (e.g. difference in food, language, climate; see Geeraret and Demes 2013 for a further review).

While studies have found support for perceived cultural distance, others have not. Forster (1997) found that sojourners from similar cultures (e.g., Americans in Britain) were just as likely to report adjustment problems as expatriates from cultures of greater distance. Western expatriates in China recognized cultural differences, but it had no impact in their adjustment (Selmer 2006). In the present study, we intend to investigate this relationship, with recent educational sojourners. In light of Selmer (2006) similar research investigation we expect to see differences between culturally close groups (Asians in China) and distant groups (non-Asians in China).

## Adaptation

Researchers have emphasized the link between acculturative stress, coping and adjustment (Berry 1997, 2006; Kuo 2013). Cervantes and Castro (1985) proposed a stress-coping-adjustment model in which coping acts as a mediator in stress-adaptation. However, studies like Aldwin (1994) and Zeidner and Endler (1996) have found that primary coping and secondary coping moderate the relationship between stress and adjustment outcomes (e.g. anxiety, depression, somatic symptoms). More recent studies by Crockett et al. (2007) and Noh and Kaspar (2003) found that primary coping buffered the negative effects of stress on psychological outcomes. In addition, Jose and Schurer (2010) found ethnicity moderated the relationship coping and maladjustment. With three distinct cultures (European and Asian New Zealanders, and those of Māori ancestry), coping differed by cultural groups across levels of individualism and collectivism. In a study of East Asians international students adapting to the United States, Cross (1995) also found primary coping (direct coping, in her words) and culture-orientation had a moderation effect on perceived stress. It was found that, for Asians students in a Western context, direct coping led to lower levels stress. On the other hand, secondary coping has been shown to have an exacerbating effect. In a study by Jose and Huntsinger (2005), teens (Asian and European American) who used secondary coping during times of high stress reported higher levels of negative adjustment. Secondary coping has been found to buffer the negative effects of stress on health related adjustment in experimental settings (Connor-Smith and Compas 2004). These studies indicate that coping (primary and secondary) induces buffering or exacerbates the effects of stress on maladjustment depending on the ethnic groups, situations and cultural contexts. While coping can also mediate stress and adaptation, we intend to only examine moderation effects between different sojourner groups in an Asian context.

## Sociocultural adaptation as outcome variable

Searle and Ward (1990) proposed the term sociocultural adaptation to describe one’s behavioral and social competence in an ability to “fit in” during cross-cultural adjustment. Sociocultural adjustment, or behavioral adaptation, has been found to increase as one acculturates and mostly follows a J-shape curve of adjustment (Geeraert and Demoulin 2013). Although behavioral adaptation tends to be most difficult at the time of arrival, it has been shown to potentially reach a ‘ceiling effect’ between 6 months and 1 year (Ward et al. 2001). Socio-cultural adaptation has been viewed as an important outcome or indicator of acculturation; yet to our knowledge, no study has investigated it within the stress-coping paradigm (see reviews by Berry 2006; Kuo 2014; Ward 2004).

## Present study

We plan to examine the role of coping (primary and secondary) on acculturative stress and sociocultural adaptation (SCA) longitudinally in educational sojourners (i.e. international students). As we recognize the complexity of the acculturative stress-coping adjustment relationship by ethnicity, we attempt to ‘unpack’ differences between cultural groups, investigate the linkage of coping and ethnicity in the stress-sociocultural adaptation relationship, and to further understand how primary and secondary coping strategies benefit socio-cultural adaptation.

In the present study, we have developed four sets of hypotheses. For educational sojourners in China, acculturative stress will be negatively related to SCA (H1a) as suggested by Berry (1997) and Ward et al. (2001). Given that primary coping is an active function, we expect primary coping to be positively correlated with SCA (H1b) and negatively correlated with secondary coping (H1c). We also hypothesize (H1d) that cultural distance will be negatively related to SCA (greater cultural distance will have lower SCA, as in Yu 2010).

Between-group analyses will be conducted and we hypothesize that non-Asians will use significantly more primary coping than Asians (H2a). Asians in China will use more secondary coping compared to non-Asians (H2b) in concordance with Chun et al. (2006), Heppner (2008), and Lam and Zane (2004). With the Cultural Distance hypothesis in mind, we expect non-Asians will report greater perceived cultural distance (H2c) and have more acculturative stress (H2d) as suggested by (Berry 1997).

Moderation analyses will be performed to determine if group (origin) in-conjunction with primary (H3) or secondary coping (H4) will function as moderators in the stress-sociocultural adaptation relationship. Previous researchers (Cohen and Wills 1985; Jose and Huntsinger 2005; Yoo and Lee 2005; Jose and Schurer 2010) have suggested that ethnicity and coping induce buffering and exacerbating effects of stress on outcomes. We will attempt to test the effects of origin and coping in separate three-way interactions: Stress × Primary × Group and Stress × Secondary × Group. We propose primary coping will buffer the negative effects of stress on SCA for non-Asians (H3a). Primary coping will exacerbate the negative effects of stress on SCA in Asians (H3b) as noted in (Jose and Huntsinger 2005, Thurber and Weisz 1997). We also expect non-Asians who use high levels of secondary coping will have deteriorating negative effects of stress in SCA (H4a), while Asians who use secondary coping will have positive effects on stress in SCA (H4b) as suggested (Yoo and Lee 2005).

## Method

### Participants

In the Fall of 2013, 121 international students at four universities in a second-tier city^2^ in Eastern China participated in the longitudinal study. Participants came from 24 countries on four continents. Other demographic variables included: Age, gender, length of residence (reported in years, months, days; “how long have you lived in China?”), and Chinese proficiency (rated 1 [poor] to 5 [excellent/fluent]; “how would you rate your Chinese language proficiency?”). Demographic details are shown in Table 1.

**Table 1:** Demographic variables

	Asian (n = 52)		Non-Asians (n = 69)		Total (N = 121)	
	Mean	SD	Mean	SD	Mean	SD
Time of residence^a^	199	345	112	175	148	261
Age	24.91	7.2	21.85	3.27	23.13	5.5
Gender	Male: 23	46 %	Male: 34	46 %	Male: 57	49 %
	Female: 29	54 %	Female: 35	54 %	Female: 64	51 %
Chinese language^b^	Beginner	31	Beginner	42	Beginner	73
	Intermediate	17	Intermediate	19	Intermediate	36
	Advanced	4	Advanced	8	Advanced	12

^a^Time of residency in days

^b^Chinese language was self-reported

## Materials

Aside from demographic questions summarized in Table 1, the questionnaire included several measures pertaining to cross-cultural adjustment (e.g. psychological symptoms, acculturation orientations, and intercultural sensitivity). However, for the present investigation, we are interested in the following variables: perceived cultural distance, stress, primary coping, secondary coping and sociocultural adaptation (measured only at time two).

## Perceived cultural distance

The perceived cultural distance (PCD) measure derived from Galchenko and van de Vijver (2007) consisted of 22 items using a seven point likert scale 1 (*very different*) to 7 (*very similar*). For example, “How similar or different do you find the food in China?” Scores were then reversed to provide a logical interpretation 1 (very similar) to 7 (very different). The scale had an internal reliability of Cronbach’s α = 0.90.

## Acculturative stress

The acculturative stress scale derived from Jose et al. (1994) consisted of 36 items detailing recent events that might have been experienced within the last month and asking how stressful they were on a scale of 0–3, where *0 implied: none (no problem or stress) and 3 implied: a lot (a big problem or high stress).* Sample scenarios included communicating, eating unfamiliar food, getting lost in the city, being separated from family and friends back home, being sick, problems with accommodation, making friends, or being unable to afford the living costs. One item from Jose et al. (1994) original scale had been removed because it pertained to speaking with an English accent. The recent events stress scale had an internal reliability of Cronbach’s α = 0.88.

## Primary and secondary coping

The coping mechanisms scale derived from Carver et al. (1989) consisted of eight items measuring primary coping (active and planning) and eight items measuring secondary coping (positive growth reinterpretation and acceptance). Primary coping constructs (active and planning) were selected because they entail a behavioral change to actively modify a negative situation or unpleasant event, thus serving as a measure of primary coping (Ward 2004). Secondary coping (positive growth reinterpretation and acceptance) constructs were chosen as they imply the internal adjustment of one’s self to the environment and/or cognitive reframing (Morling and Evered, 2006). Items were rated *1* = *don’t do this at all* and *4* = *do this a lot*. Example items include “I take direct action to get around my problem” (active), “I try to come up with a strategy about what to do” (planning), “I accept that this has happened and that it can’t be changed,” (acceptance) and “I try to grow as a person as a result of an experience” (positive reinterpretation and growth). Overall, primary coping and secondary coping had Cronbach’s alpha internal reliabilities of 0.84 and 0.77 respectively.

## Sociocultural adaption measured at time 2

The Sociocultural Adaptation Scale Revised (SCAS-R) derived from Wilson and Ward (2010) consisted of 21 items that measured competency in living in a new culture measured on a 5-point scale ranging from 1 (not very competent) to 5 (very competent). An example item is “Adapting to the population density.” The scale’s internal reliability was Cronbach α = 0.88.

## Procedure

During the first week of classes in the Fall of 2013, international students were asked to participate in a research study. The pen and paper questionnaire took approximately 20–30 min to complete and researchers were present to answer any questions. Questionnaires were available in three languages (English, Mandarin Chinese and Korean). Chinese and Korean versions were translated by bilingual colleagues and then back translated into English by a separate translator to check accuracy of survey translation. The majority of the students completed the questionnaire in English (80 %) while all participants were free to choose the language they felt most confident in. There was no relationship or any differences between core variables and language they chose to complete at either survey time.

The paper survey included a code (students’ initials and last four digits of the student’s ID card) to match participants from time one and time two. 226 international students agreed to participate in the study, as they knew it had no impact in their course or official standing with the university. Before the end of the semester, researchers returned to administer the second questionnaire. Overall, 124 students completed the second questionnaire. Respondents who participated in both questionnaires received a small gift (valued approximately at $1).

## Groupings

Due to the distribution of our sample, we based our group clusters on intraregional migration (Asians) vs. intercontinental migration (non-Asians) (as explained in Rystad 1992). Our groupings consisted of: (1) Asian group (*n* = 52), primarily represented by countries from East Asia (e.g. Japan, and South Korea) and Southeast Asia (e.g. Thailand, Vietnam, Indonesia and Malaysia); (2) Non-Asian group (*n* = 69), represented from countries in Europe (e.g. Italy, France, Germany and Britain) and North America (e.g. Canada and United States) and a small sample of Africans (*n* = 17) from West and East Africa (e.g. Nigeria, Ghana, Congo, Zimbabwe). We decided to include the African sample with the otherwise “western” group due to the small sample size and similarity in perceived cultural distance scores. Chinese scholars in Mandarin language acquisition (Gao et al. 2012) often divide groups based (e.g. Asian vs non-Asian) on historical interactions as documented by Chinese influence in Asia.

## Analyses strategy

We followed previous researchers who conducted similar cross-cultural adjustment studies on international students in New Zealand (Jose et al. 2007) and Russia (Suanet and Van de Vijver 2009) by first addressing attrition, then exploring our correlation findings of the main variables. We then tested for group-differences using a multivariate analysis of variance (MANOVA) analysis. Finally, we examined our main effects and moderation hypotheses using hierarchical linear regression analysis.

## Results

### Preliminary analyses

As mentioned above, 124 participants completed the both time waves for a respectable response rate of 54 %, but due to missing items, 3 respondents were excluded leaving 121 participants for analyses. In order to examine possible selection bias due to sample attrition, *t* tests analyses were run to compare participants who completed both time waves and those who only completed time one questionnaire. We examined attrition rates on demographic factors (age, groups, Chinese language, time in China) and principal variables (stress, primary, secondary coping and perceived cultural distance), but no significant differences were found: Age (*t(*226) < 1); Mandarin proficiency (*t*(226) = −1.60, *p* > 0.05); time of residence (*t*(226) = −1.52, *p* > 0.05); stress (*t*(226) = 1.124, *p* > 0.05); primary coping (*t*(226) < 1); secondary coping (*t*(226) < 1); and perceived cultural distance (*t*(226) < 1).

After controlling for demographic variables (gender, age, time in China and Chinese language), correlation analyses revealed a moderate to strong relationship between primary coping and secondary coping (*r* = 0.56, *p* < 0*.01*). Primary coping was also positively related to PCD (*r* = 0.19, *p* < 0.05) and negatively related to stress (*r* = −0.23, *p* < 0*.01*). Secondary coping was positively related to PCD (*r* = 0*.14, p* < 0.05) and also negatively to acculturative stress (*r* = -0*.21, p* < 0.05). Interestingly, acculturative stress was unrelated to SCAS, therefore rejecting H1a. Primary coping and secondary coping were both positively related to SCAS (*r* = 0*.17 p* < 0.05) and (*r* = 0*.21, p* < 0.05), thus confirming H1b, while rejecting H1c. Perceived cultural distance was unrelated to SCAS (*r* = 0.03, *ns*), therefore rejecting H1d.

In accordance with previous research exploring cross-cultural adaptation of difference groups (see Galchenko and van de Vijver 2007; Jose et al. 2007; Suanet and Van de Vijver 2009), we conducted correlations on core variables between our subsamples (see Table 2). We also conducted a MANOVA (Group × Gender) for our between-groups analysis to look for significant effects on all variables of interest (independent variables: PCD, stress and primary, secondary coping and dependent variable: SCAS).

**Table 2:** Correlations of main variables by group

	1	2	3	4	5
1. Primary	–	0.42***	0.01	0.11	0.05
2. Secondary	0.63***	–	0.21	0.13	0.19
3. PCD	0.12	−0.08	–	0.03	0.03
4. Stress	−0.46**	−0.38**	−0.04	–	0.21
5. SCAS	0.24	0.18	−0.06	−0.33*	–

Correlations in the non-Asian subsample are presented above diagonal

Correlations in the Asian subsample are presented below diagonal

Controlled for Age, Gender, Chinese language and time in China

* p ≤ 0.05; ** p ≤ 0.01, *** p ≤ 0.001

The results indicate a significant effect by gender, Wilk’s λ = 0.77, *F*(5,113) = 6.70, *p* < 0.001 η^2^ = 0.23. Moreover, there was a significant effect by ethnicity, Wilk’s λ = 0.79, *F*(5,113) = 6.03, *p* < 0.001 η^2^ = 0.21. We obtained a marginally significant group by gender interaction, Wilk’s λ = 0.92, *F*(5,113) = 2.13, *p* < 0.07, η^2^ = 0.09.

Differences between groups were analyzed by means of between-subjects tests (see Table 3). Non-Asians used more primary coping (*M* = 2.86, *SD* = 0*.54*) than Asians (*M* = 3.12, *SD* = 0*.53), F*(1,120) = 6.19, *p* = 0.014, η^2^ = 0.05, thus confirming H2a. Surprisingly, Asians reported lower secondary coping (*M* = 2.85, *SD* = 0*.65*) compared to non-Asians (*M* = 3.2, *SD* = 0*.44*), *F*(1,120) = 14.74, *p* = 0.00;η^2^ = 0.11, thus rejecting hypothesis 2b. In addition, Asians reported lower PCD (*M* = 4.96 *SD* = 0.83) compared to non-Asians (*M* = 5.38, *SD* = 0*.57*), *F*(1,120) = 10.00, *p* = 0.002, η^2^ = 0.08., thus confirming hypothesis 2c. SCAS was also statistically significant *F*(1,120) = 10.67, *p* = 0.001, while acculturative stress was not (*F*(1,120) = 1.37, *p* = 0.25), thus rejecting hypothesis 2d.

**Table 3:** Mean scores between groups

	Groups				All (N = 121)	
	Asians (n = 52)		Non-Asians (n = 69)			
Scale	M	SD	M	SD	M	SD
PCD	4.96	0.83	5.38**	0.57	5.20	0.72
Primary	2.86	0.54	3.12**	0.53	3.01	0.54
Secondary	2.85	0.55	3.2**	0.44	3.05	0.52
Stress	18.31,	10.49	16.35	5.88	17.19	8.2
SCAS	3.01	0.62	3.37***	0.62	3.21	0.63

* *p* < 0.05, ** *p* < 0.01, *** *p* < 0.001

Between-subjects comparison also found significant gender differences. Males (n = 57) reported greater use of primary coping (*M* = 3.19 *SD* = 0*.49)* compared with females (*M* = 2.84 *SD* = 0*.54*), *F*(1,120) = 15.29, *p* < 0.001, η^2^ = 0.12. Males also reported great use of secondary coping (*M* = 3.19 *SD* = 0*.49)* compared with females (*M* = 2.84 *SD* = 0*.54*), *F*(1,120) = 4.44, *p* < 0.04, η^2^ = 0.04. Surprisingly, results indicated that females (n = 64) reported higher stress (M = 18.82 *SD* = *8.72)* compared with males (M = 15.35, *SD* = *7.22*), *F*(1,120) = 5.53, *p* = 0.002, η^2^ = 0.05. Females also reported great PCD (*M* = 5.45, *SD* = 0*.89)* compared with males (*M* = 4.94, *SD* = 0*.80*) *F*(1,120) = 18.46, *p* < 0.001, η^2^ = 0.14. The number of significant main effects made it necessary to co-vary gender in our moderation analyses (as conducted in Yoo and Lee 2005).

## Results of moderation analyses

A hierarchical multiple regression analysis was implemented to test the interaction effects on our dependent variable, SCAS. Our three-way interactions were: Stress × Primary × Group (H3) and Stress × Secondary × Group (H4).

The following procedure was used for both regressions tests. Step 1: control variables^3^ (Gender [female 0, male 1], living time in China, and Chinese language), Step 2 and 3: our independent variables (Stress and Primary/Secondary Coping), Step 4: Group (dummy coded 0 = non-Asian, 1 = Asian), Step 5: all combinations of two-way interactions were included (Stress × Coping; Stress × Group, Coping × Group) and Step 5: three-way interaction term. To reduce the likelihood of multi-collinearity, all independent variables, moderators, and interaction variables were mean-centered (Aiken and West, 1991). Results are presented in Tables 4 and 5.

**Table 4:** Hierarchical multiple regression analysis testing groups with primary coping as moderator of stress

Predictor	Step 1				Step 2				Step 3				Step 4				Step 5				Step 6			
	Δ*R* ^2^	Β	*Beta*	*p*	Δ*R* ^2^	Β	*Beta*	*p*	Δ*R* ^2^	Β	*Beta*	*p*	Δ*R* ^2^	Β	*Beta*	*p*	Δ*R* ^2^	Β	*Beta*	*p*	Δ*R* ^2^	Β	*Beta*	*p*
Step 1	0.02																							
Constant		3.10		0.00		3.10		0.00		3.03		0.00		3.18		0.00		3.24		0.00		3.17		0.00
Gender		−0.11	−0.09	0.32		−0.10	−0.08	0.41		−0.03	−0.02	0.81		−0.04	−0.03	0.76		−0.02	−0.02	0.85		0.03	0.03	0.79
CN language		0.12	0.13	0.16		0.12	0.13	0.19		0.13	0.14	0.13		0.12	0.13	0.16		0.08	0.09	0.33		0.10	0.11	0.23
Time in China		−0.00	−0.00	0.94		0.00	0.01	0.96		0.00	0.01	0.96		0.00	0.05	0.63		0.00	0.07	0.45		0.00	0.07	0.44
Step 2					0.00																			
Stress						0.00	0.05	0.58		0.00	−0.02	0.86		0.00	0.01	0.93		−0.01	−0.16	0.37		−0.01	−0.04	0.81
Step 3									0.04*															
Primary Coping										−0.24	−0.20	0.04		−0.17	−0.14	0.14		−0.10	−0.09	0.46		−0.11	−0.10	0.41
Step 4													0.06**											
Group														−0.32	−0.26	0.01		−0.27	−0.21	0.03		−0.32	−0.26	0.01
Step 5																	0.05							
Stress × ggroup																		0.01	0.08	0.66		0.01	0.13	0.50
Primary × group																		−0.10	−0.06	0.65		−0.08	−0.04	0.73
Stress × primary																		0.03	0.23	0.04		0.08	0.61	0.00
Step 6																					0.05**			
Stress × Primary × group																						−0.08	−0.53	0.01
Total *R* ^*2*^	0.02				0.03				0.06				0.12				0.18				0.23			

* N* = 121; CN Language = Chinese Language; Stress = Acculturative Stress; Dependent Variable is Sociocultural Adaptation

* p < 0.05. ** p < 0.01

**Table 5:** Hierarchical multiple regression analysis testing groups with secondary coping as moderator of stress

Predictor	Step 1				Step 2				Step 3				Step 4				Step 5				Step 6			
	Δ*R* ^2^	Β	*Beta*	*p*	Δ*R* ^2^	Β	*Beta*	*p*	Δ*R* ^2^	Β	*Beta*	*p*	Δ*R* ^2^	Β	*Beta*	*p*	Δ*R* ^2^	Β	*Beta*	*p*	Δ*R* ^2^	Β	*Beta*	*p*
Step 1	0.02																							
Constant		3.09		0.00		3.09		0.00		3.01		0.00		3.14		0		3.18		0.00		3.14		0.00
Gender		−0.11	−0.09	0.32		−0.10	−0.80	0.41		−0.05	−0.04	0.65		−0.05	−0.04	0.64		−0.05	−0.04	0.67		−0.04	−0.03	0.72
CN Language		0.12	0.13	0.16		0.12	0.13	0.19		0.15	0.17	0.07		0.14	0.15	0.11		0.11	0.12	0.20		0.12	0.13	0.16
Time in China		−0.00	−0.01	0.94		0.00	0.01	0.96		0.00	0.01	0.94		0.00	0.04	0.66		0.00	0.07	0.47		0.00	0.07	0.45
Step 2					0.00															.				
Stress						0.00	0.05	0.58		0.00	0.00	0.99		−0.00	−0.00	0.99		−0.01	−0.18	0.29		−0.01	−0.14	0.41
Step 3									0.08**															
Secondary coping										−0.34	−0.29	0.00		−0.26	−0.21	0.03		−0.32	−0.26	0.06		−0.35	−0.29	0.04
Step 4													0.04*											
Group														−0.27	−0.22	0.02		−0.26	−0.20	0.04		−0.29	−0.23	0.02
Step 5																	0.03							
Stress × Group																		0.02	0.16	0.39		0.02	0.23	0.23
Secondary × Group																		0.14	0.08	0.58		0.23	0.14	0.35
Stress × Secondary																		0.02	0.14	0.22		0.06	0.44	0.02
Step 6																					0.03*			
Stress × Secondary × Group																						−0.06	−0.06	0.05
Total *R* ^*2*^	0.02				0.03				0.10				0.14				0.17				0.20			

* N* = 121; CN Language = Chinese Language; Stress = Acculturative Stress; Dependent Variable is Sociocultural Adaptation

* p < 0.05. ** p < 0.01

The overall model revealed three significant results and explained 23 % of variance *F(10, 110* = *7.36, p* < 0.01). In Step 3, group was significant (β = −0*.26, p* < 0.01) and explained 6 % of the variance. While interactions between primary and stress in step 4 (standardized β = 0*.61, p* < 0.001) and Primary × Stress × Group in step 5 (standardized β = −0.53, *p* < 0*.01*) together explained 10 % of the variance of socio-cultural adaptation. Finally, hypothesis 3 was confirmed; the three-way interaction between Stress × Primary × Group on SCAS was significant, (standardized β = −0.53, t = −2.71, *p* = 0.01).

As suggested by researchers (Cohen and Cohen, 1983; Cohen and Wills 1985; Jose and Huntsinger 2005), a three-way interaction can be graphed for interpretation. We followed procedures using Modgraph (Jose, 2002) and graphed the three-way interaction to depict the moderation effect. Simple slope analysis for Asians show: low level primary coping: *t*(49) = 1.75, *p* = 0.09; medium: *t*(49) = 0.35, *ns.*; high: *t*(49) = −1.11, *ns*. For non-Asians, simple slope analysis for three conditions yielded significant findings for two slopes: low: *t*(66) = −3.08, p < 0.01, medium: *t*(66) = −0.15, *ns*., and high: *t*(66) = 1.99, *p* < 0.05.

**Fig. 1:**
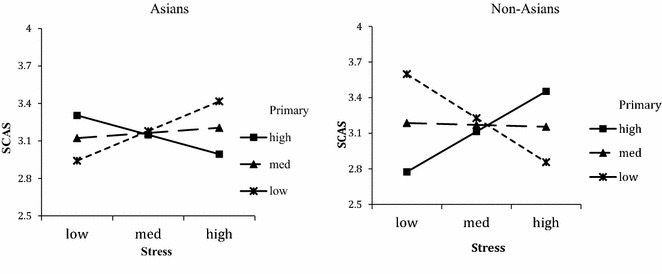
Moderation effects of primary coping on stress and socio-cultural adaptation

As shown in Fig. 1, primary coping exacerbates the negative effects of stress on socio-cultural adaptation for Asians. Stress leads to higher levels of SCA only under the condition of low primary coping. This finding confirms hypothesis 3a. As for non-Asians, primary coping buffers the negative effects of stress on socio-cultural adaptation, thus confirming hypothesis 3b. Under low levels of primary coping, stress leads to a deleterious socio-cultural adaptation, however under high levels of primary coping it leads to better SCA.

In our second moderation analysis (Stress × Secondary × Group), we repeated the statistical procedure using secondary coping in place of primary coping. The overall model accounted for 20 % of the variance of socio-cultural adaptation after 90 days *F*(10, 110) = 3.81, *p* = 0.05). In step 3, secondary coping (standardized β = −0.29*, p* = 0.04***) and group (standardized β = −0.23*, p* = 0.02) revealed significant effects, suggesting that secondary coping is a deleterious strategy for acculturative stress on sociocultural adaptation of non-Asians in China. Interactions between stress and secondary in step 4 (standardized β = 0.44*, p* = 0.02), as well as Stress × Secondary × Group in step 5 (standardized β = −0.40*, p* = 0.05), explained 6 % of variance of SCAS. The three-way interaction between Stress × Secondary × Group on SCAS was significant, thus confirming H4 (standardized β = −0*.06*, *t* = −1.95, *p* = 0.05).

The three-way interaction was then depicted using Modgraph (Jose, 2002). Simple slope analyses were performed on each group and show: Asians low secondary: *t*(49) = 1.80, *p* = 0.08; medium: *t*(49) = 0.91, *ns*; high: *t*(49) = −0.29, *ns*., and non-Asians low secondary: *t*(66) = −2.42 *p* < 0.02; medium: *t*(66) = −0.78, *ns,* and high: *t*(66) = 0.97, *ns.*

**Fig. 2:**
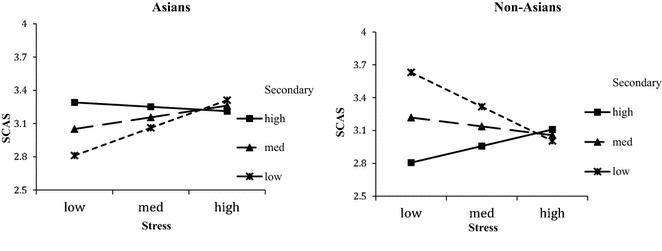
Moderation effects of secondary coping on stress and socio-cultural adaptation

As shown in Fig. 2, secondary coping has positive benefits for reducing acculturative stress and increasing socio-cultural adaptation for Asians. However, secondary coping appears to be an effective strategy for attenuating low and medium levels stress, thus partially supporting H4a. For non-Asians, secondary coping worsens the negative effects of stress on sociocultural adaption. In high stress contexts, secondary coping has minimal benefits for non-Asians, thus supporting H4b.

In summary, for both groups secondary coping is an effective strategy reducing stress across low and moderate levels. In high stress situations, however, non-Asians benefit from primary coping, while Asians have deleterious effects from high levels of primary coping.

## Discussion

This study explored the predictive nature of acculturative stress, primary, and secondary coping on sociocultural adaptation 90 days later in an under researched population in sample. The ultimate purpose of this study was to reveal cultural differences in how Asians and Non-Asians utilize primary and secondary coping strategies and how they specifically benefit from behavioral adjustment. Below we will discuss specific results in detail.

Contrary to the proposed hypotheses, acculturative stress, and perceived cultural distance had no relationship with SCA. Furthermore, perceived cultural distance did not correlate with acculturative stress. It was, however, interesting that both primary and secondary coping correlated with SCA. These results suggest that even though individuals may expect cultural distance and experience acculturative stress, it may not have a direct relationship with sociocultural adaptation 90 days later. In actuality, this finding supports the coping framework intertwined in acculturation research and our research has confirmed the relationship between coping, stress and sociocultural adaptation. Surprisingly, secondary coping had a slightly stronger relationship with SCA, which suggests that all ethnic groups may also benefit from the use of secondary coping controls.

As expected, Asians reported lower perceived cultural distance compared with Non-Asians, but this group did not have higher sociocultural adaptation. This supports recent critics like Kashima and Abu-Rayya (2014), Geeraert and Demoulin (2013), and Suanet and Van de Vijver (2009) who have failed to find support for cultural distance across large data sets using objective cultural distance measures. The cultural distance hypothesis deserves further attention across multi-national and multi-ethnic samples.

Further group comparisons revealed that non-Asians used more primary coping compared to Asians. Contrary to our hypothesis, secondary coping was also used to a greater extent by non-Asians. This finding partially supports previous literature (McCarthy et al. 1997; Selmer 2002), but still deserves further attention. According to research by Chun et al. (2006) and Heppner (2008), Asians may also use other collective coping strategies such as: family support, respect, intracultural coping, relation universality, forbearance social activity and fatalism (as in Sinha and Watson 2007). Given the complexity of ‘collective coping’, Asians and other minority groups acculturating are still under researched and deserve further investigation.

Though not a principal focus of this study, the gender differences found suggest that female exchange students in China experience greater acculturative stress and lower usage of primary and secondary coping. While it is sometimes reported that women may be at greater risks during acculturation, results are often mixed regarding stress and coping (Beiser et al. 1988, Ward et al. 2001). In some cases, men report poorer adjustment (Boski 1990). In a study by Lee and Padilla 2014, researchers investigated stress and coping of Korean students, finding that men reported greater acculturative stress noting language and cultural differences as the source of greatest trouble. Berry (1997) points out that it is problematic to generalize gender in acculturation studies; instead, it is necessary to compare the difference in gender roles between the host culture and home culture of the sojourner. Returning to the Chinese context, Bond (2010) suggests gender inequality may stem from patriarchal Confucian values. It is possible that female sojourners in China may experience some gender discrimination or societal pressure from the mainstream culture, thus hindering their overall adjustment. However, it is beyond the scope of the present study to comment strongly on the attitudes towards and role of women in modern Chinese society.

The most noteworthy finding in the present study is the moderation effects of group with primary and secondary coping in the stress-sociocultural relationship. These findings clearly depict how the sociocultural adaptation of different ethnic groups may benefit from or be harmed by certain coping strategies when handling acculturative stress. Firstly, primary coping appears extremely effective for Non-Asians. Low levels of primary coping in high stress situations result in lower socio-cultural adjustment, while high primary coping has great benefits. To our knowledge, this is the first study to explore this relationship. In fact, one would expect primary coping (being active and behavioral in nature) to be highly correlated with one’s ability to fit in socially. Unfortunately for Asians, higher levels of primary coping tend to increase the negative effects of stress thus resulting in lower levels of socio-cultural adjustment. According Kuo (2014), social support, secondary, and collective coping may be the preferred and effective strategies for Asians. Chun et al. (2006) noted that collectivistic societies use less primary coping and it may be detrimental for certain groups to use primary coping. The present findings support this and further show that primary coping may not be a beneficial strategy for Asians in the sociocultural context.

While secondary coping tends to be beneficial in low and medium stress situations for both Asians and non-Asians, it is still unclear as to its benefits in terms of socio-cultural adjustment. Jose and Huntsinger (2005) found emotion focused (i.e. secondary) coping to be detrimental for American (of both Asian and European descent) students in high stress situations. Our findings support this notion. For Asians, low levels of secondary coping were associated with better adjustment. However, in high stress situations, secondary coping is neither harmful nor helpful. We suspect a third type of coping strategy to be in play here, possibly social support. In high stress situations, Asians may seek support (either emotionally or instrumentally) from friends or family.

Another possible explanation comes from Selmer and Shiu (1999) who discovered ‘culturally close’ Hong Kong Chinese business managers working in Mainland China experienced frustration, resentment and in turn withdrew socially from mainland society. It is possible that Asians in China may undergo some perceived discrimination and not employ primary coping strategies. Asians adapting to living in an Asian context may prefer to use avoidant or collective coping strategies as a way to avoid “rocking the boat” (as explained in Kuo 2014). Björkman and Schapp (1994) revealed that Mainland Chinese despised and/or envied overseas Chinese. The interpretation of this result should be cautioned and studied more to reveal more concrete evidence.

As for non-Asians, primary and secondary coping is beneficial and the results in Figs. 1 and 2 show a complete picture of how non-Asian international students should use coping in order to improve adjustment. In low and medium stress situations, low and moderate secondary coping is beneficial, while high primary coping is more beneficial in high stress situations.

It is also possible that the above results may also be entirely due to the apparent difference between the sojourners. In an Asian context, non-Asians (Westerners and Africans) have completely different appearance from the locals. Thus, non-Asians may experience some social handicap as being an obvious ‘foreigner,’ whereas non-Chinese Asians could potentially be mistaken as Chinese and be expected to behave as such. This deserves further attention by researchers in Asia.

## Conclusions

This study is likely the first investigation to fully explore a stress-coping-sociocultural adaptation model outside of the Western world. As suggested by researchers (Kuo 2013; Chun et al. 2006; Sinha and Watson 2007), more studies need to explore stress and coping on various migrant populations across different host cultures. Expanding on this, our study also explored an under researched group of educational sojourners that have seen tremendous growth in recent years (see Belyavina 2013). From a global perspective, the People’s Republic of China is making great advances in internationalizing education and acculturation research needs to continue to explore this area to better support and facilitate smooth cross-cultural transitions for all sojourners (both seeking economic and educational advances). Last, but not least, acculturation research has expressed the need for understanding changes during adaptation and the best way to do that is with longitudinal studies (Berry 1997 and 2007). In the present study, we employed a longitudinal design that measured the predictability of socio-cultural adaptation as an outcome variable. While cross-sectional designs are limited in this regard, our study was able to reveal the importance of primary and secondary coping as a way to buffer acculturative stress and enhance behavioral adaptation for certain ethnic groups.

With this in mind, one major limitation is that socio-cultural adaptation was only measured at time 2. Unfortunately, this study lacks the ability for acculturative stress, primary and secondary coping to predict increments in sociocultural adaption over time. More specifically, given that questionnaires were not administered at the time of arrival, it is possible some of the international students experienced some SCA prior to the time of the research investigation.

Another major shortcoming is that the data were self-reported and questionnaires were available in only three languages (Korean, Mandarin and English). To fully accommodate all exchange students in China, it would be of great interest to provide other language options (see Demes and Geeraert 2013), adding Russian, Spanish, and French as a minimum. In addition, English proficiency should be controlled in order to reduce the possibility of confounding variables when interpreting the questionnaire.

Another recognizable limitation is with the proposed group clusters, specifically merging the African and Western sample into the group clustered as “non-Asian”. It is of great importance to caution researchers to examine these results. As with any research study investigating cultural differences, the presented results may be a main effect of culture confounded with a language or response bias. To be more specific, we must raise concerns of possible response acquiescence. Certain Asian groups are subject to following the Confucian norm of harmony, which could result in differences in self-responses in surveys compared to individualistic societies (as explained in Selmer 2002). This must be considered when carefully interpreting the results of this study.

Furthermore, our sample was a diverse group of educational sojourners studying Mandarin Chinese and graduate degrees; unfortunately it was not possible to capture the sample over a longer period of time or in multiple cities across China. A 3-month interval was chosen due to semester school calendar, while taking into consideration many international students may return home for winter holidays (e.g. Christmas, New Year, and Chinese New Year). It would be of great interest to administer surveys on arrival and follow them throughout the duration of their study in China (as conducted in Ward et al. 1998, and Geeraert and Demoulin 2013). To reiterate, this is another major limitation as students’ acculturative stress, coping, and adaptation could all have fluctuated over the course of the research study and could account for some variance in the results.

Despite these limitations, the present research investigation possesses some significant contributions. It appears that cultural distance may not always be a principal factor in adjustment or instead may truly depend on the host culture or pre-departure expectations. In fact, culturally distant groups reported greater adjustment; even though there was cultural dissimilarity, individuals may still adjust smoothly. As a result, stress-coping-adjustment gains great support. This study proposes that non-Asians benefit from primary and secondary coping, while Asians may have deleterious effects from using high levels of primary coping. Asians also benefit from low to moderate usage of secondary coping, however in high stress-induced situations secondary coping may not be the desired coping strategy.

In general, our research provides implications for coping researchers to further explore cross-cultural comparisons of international students at university campuses and in the workplace. Furthermore, this study supports the inability to generalize Euro-American coping strategies for distinct cultures around the world (Kuo 2013; Chun et al. 2006). In fact, this study further demonstrates the complexity of coping calling for research to consider ‘collective coping’ and the role of social support for Asians.

In closing, this study has important implication for higher education institutions in China with international students, as this study is one of the first to focus on Mainland China. With studies like this one, universities will hopefully be able to better support various students with effective stress management.

## Footnotes

1 Individualism/collectivism is one of the four major dimensions measuring cultural distance (Hofstede 1980). Tremendous amount of research has strongly correlated individualism and collectivism with standard concepts of comparing Western/Eastern societies (see the review by Ronen and Shenkar (2013). Chinese cities are ranked by GDP output. Outside of the four first-tier metropolises of Beijing, Shanghai, Guangzhou, and Shenzhen, second-tier cities are large (8-digit populations) and relatively prosperous. Age was not included in these regression analyses. In preliminary analysis, it was found to be strong predictor in regression results and correlational findings. In fact, results indicate that sociocultural adaptation may be easier for younger students (after controlling for demographics: age correlates with SCAs at *r* = −0.32, *p* < 0.001 and a standardized β = −0.32, *p* < 0.001). To further expand on this, most of the international students had never been overseas (33 %) and 44 % (n = 53) had only traveled abroad. It is then possible that younger participants experienced a “first time effect” (see McKeown 2009) where they were more open to adjusting and integrating into Chinese culture. Age should be examined more closely in acculturation research.
